# Molecular and genetic characterization of emerging carbapenemase-producing *Acinetobacter baumannii* strains from patients and hospital environments in Bangladesh

**DOI:** 10.1016/j.infpip.2022.100215

**Published:** 2022-04-27

**Authors:** Refath Farzana, Göte Swedberg, Christian G. Giske, Badrul Hasan

**Affiliations:** a Section for Infectious Diseases, Department of Medical Sciences, Uppsala University, Uppsala, Sweden; b Department of Medical Biochemistry and Microbiology, Uppsala University, Uppsala, Sweden; c Department of Medical Microbiology, Institute of Infection and Immunity, Cardiff University, UK; d Department of Laboratory Medicine, Division of Clinical Microbiology, Karolinska Institutet, Stockholm, Sweden

**Keywords:** *A. baumannii*, Antibiotic resistance, Environment, Patients, Hospital, Bangladesh

## Abstract

**Background:**

Carbapenemase-producing multidrug-resistant (MDR) *Acinetobacter baumannii* is a global health care problem. MDR *A. baumannii* has emerged as an important nosocomial pathogen, costing many lives worldwide including Bangladesh.

**Aim:**

To investigate the detailed molecular epidemiology of carbapenem-resistant *A. baumannii* (CRAB) both from patients and the hospital environment, to shed light on genetic characteristics and transmission dynamics.

**Methods:**

A set of 49 clinical *A. baumannii* strains collected during early 2015 was received from the clinical microbiology laboratory of Dhaka Medical College Hospital (DMCH) in Bangladesh. Additionaly, 100 environmental samples were also collected from the hospital surfaces of Dhaka Medical College Hospital and analyzed for carbapenamase-producing *A. baumannii*. CRAB were identified by culture on selective plates, biochemical testing and MALDI-TOF. All isolates were characterized by susceptibility testing, realtime-PCRs, conventional PCR, MLST and sequencing.

**Findings:**

Clinical *A. baumannii* were resistant to ciprofloxacin (100%), imipenem (91.8%), meropenem (91.8%), gentamicin (91.8%), amikacin (87.7%), and trimethoprim-sulfamethoxazole (61.2%). The majority (59%) of the isolates were MDR. All environmental *A. baumannii* (n=10) were resistant to imipenem, meropenem, gentamicin, amikacin, and ciprofloxacin. Strains carried the following antibiotic resistant genes; *bla*_OXA-23,_*bla*_OXA-58,_*bla*_PER-7,_*qnrB1*, *qnrC1, aac(6′)1b-cr* and *armA*. A total of 36 different clones were identified by rep-PCR and common clonal clusters were found both in patients and hospital environments. MLST analysis revealed different sequence types (ST2, ST10, ST149, ST575, ST1063 and ST1065). In clinical and environmental settings. *A. baumannii* ST2 dominated in both clinical and environmental settings. Both clinical and environmental *A. baumannii* strains with known STs carried several biofilm-related genes; *bap, csuE,* and *pgaB*.

**Conclusion:**

Widespread dissemination of MDR *A. baumannii* in the DMC hospital of Bangladesh is a serious problem.

## Introduction

*Acinetobacter baumannii* is an important opportunistic pathogen that has increasingly been reported worldwide. *A. baumannii* has emerged as an important nosocomial pathogen, costing many lives worldwide [1]. Over the last decades, multidrug-resistant (MDR) *A. baumannii* has become a serious public health issue as the treatment options are extremely limited. Different antibiotic resistance phenotypes and genotypes have been reported in *A. baumannii*. For example, *bla*_*PER-7*_ is an extended-spectrum β-lactamase with increased activity toward broad-spectrum cephalosporins in *A. baumannii* [2]. The most concerning resistance mechanism in this species is production of carbapenemases such as *bla*_OXA-23_-like, *bla*_OXA-51_-like, *bla*_OXA-58_-like, and *bla*_OXA-143_-like, as well as metallo-β-lactamases (MBL), most notably the New Delhi carbapenemase *bla*_NDM_ [1,3,4]. Carbapenemase-producing *A. baumannii* has been reported globally, including in several developing countries [1,3,4]. Carbapenem resistance tends to occur in conjunction with other antibiotic resistances, such as fluoroquinolones and aminoglycosides. Several reports indicate that fluoroquinolone (e.g. *aac(6′)-Ib-cr*) and aminoglycoside (e.g. *armA*) resistance genes can be harboured on the same plasmids as those carrying genes encoding carbapenemase production [5]. In addition to antibiotic resistance, the ability to form biofilms is an important virulence factor in *A. baumannii*. Several genes are involved in biofilm formation of *A. baumannii* such as *csu* locus (encoding the chaperone –usher Csu fimbriae), *pga* locus (encoding the polysaccharide poly-N-acetyl-glucosamine), and *bap* (encoding the biofilm-accociated protein) [6]. These genes are frequently reported in clinical isolates of *A. baumannii* [6].

In recent years, an increasing frequency of MDR *A. baumannii* infection has been causing severe problems among patients admitted to hospitals in Bangladesh. Infection control practice and medical waste management in Bangladeshi hospitals is very poor or hardly practiced [7]. Only a limited number of reports have so far described the burden of carbapenem-resistant *A. baumannii* carrying MBL genes in this geographical setting, and these reports are insufficient for understanding the detailed epidemiology or for assessing the human health risks associated with the hospital environments and medical devices in Bangladeshi hospitals [8]. The aim of this study was to investigate the detailed molecular epidemiology of carbapenem-resistant *A. baumannii* both from patients and the hospital environment, to shed light on genetic characteristics and transmission dynamics. In addition, some genes related to biofilm production were investigated for.

## Methods

### Ethical permission

Ethical approval was obtained from the Ethical Committee of Dhaka Medical College Hospital (DMC/Ethical/2013-159) to perform this study.

### Sample collection and bacterial identification

In Sweden, we received a collection of 49 clinical *A. baumannii* isolates from the clinical microbiology laboratory of the Dhaka Medical College Hospital in Dhaka City, Bangladesh. All isolates were collected between January and March 2015 from 73 patients in various wards (Example: ICU, surgery, medicine, burn unit, obstetrics, and gynecology). Sources of the isolates included blood, urine, wound swab, cerebrospinal fluid, high vaginal swabs, catheter tips and tracheal aspirates. At the same time, 100 samples were collected from different environmental surfaces of the Dhaka Medical College Hospital using Amies transport medium (Sarstedt, Nümbrecht, Germany); sites sampled were bed rails, bed sheets, switchboards, sinks, blood pressure cuffs, ventilators, catheters, O2 masks, suckers, toilets, and sewage-drains. *A. baumannii* clinical isolates were cultured on CHROMagarTM Acinetobacter media. Environmental samples were enriched at 37°C overnight in Tryptic Soy broth supplemented with meropenem (0.125 mg/L), followed by inoculation onto CHROMagarTM Acinetobacter media (CHROMagar, France). Species identification was performed using biochemical tests (e.g. oxidase and catalase) followed by MALDI-TOF/MS (Bruker Daltonics, Billerica, MA).

### Phenotypic and genotypic characterization of antibiotic resistance and bioflim

All clinical and environmental strains were subjected to antibiotic susceptibility testing. EUCAST disc diffusion susceptibility testing was performed using 7 clinically important antibiotics; ciprofloxacin (5μg), imipenem (10μg), meropenem (10μg), gentamicin (10μg), amikacin (30μg) and trimethoprim-sulfamethoxazole (25μg). All discs were provided by Oxoid Ltd. (Basingstoke, Hampshire, England). *Escherichia coli* ATCC 25922 and *Pseudomonas aeruginosa* ATCC27853 were used as quality control strains. Isolates with resistance to three or more antibiotic classes were considered as MDR strains.

Genomic DNA was extracted from overnight culture by Maxwell® 16 Cell DNA Purification Kit (Promega, Madison, USA) using automated Maxwell® 16 SEV Instrument. Each extracted genomic DNA was centrifuged for 10 minutes at 13000 rpm and supernatant was stored at -20 °C for further uses.

The OXA-carbapenamase genes were screened; the presence of *bla*_OXA-23_-like, *bla*_OXA-24_-like, *bla*_OXA-58_-like, and *bla*_OXA-143_-like genes were investigated by PCR, as described previously [9,10]. The screening of MBLs genes was also performed by various real time-PCRs for *bla*_VIM_, *bla*_NDM_*, bla*_IMP_*, bla*_GIM_*, bla*_SPM_ and *bla*_SIM_ [11]. All isolates were screeined for *bla*_KPC_ using a method described previously [12]. Detection of *bla*_PER_ was performed using a method described before on selected strains [2]. The strains displaying resistance to aminoglycosides and fluoroquinolones were further tested for their respective resistance genes. To detect fluoroquinolone resistance genes (*qnrA1, qnrB1, qnrS1, qepA, qnrC1* and *aac(6′)-Ib-cr*), a series of PCRs were performed [13,14]. Screening for the presence of 16S rDNA-methylase encoding genes (*rmtA, rmtB, rmtC, rmtD, armA*) was among aminoglycoside resistant isolates by simplex and multiplex PCRs [15]. A set of strains with known MLST-types were selected to investigate for biofilm-associated genes; the presence of the biofilm related genes *bap*, *csuE*, and *pgaB* were assessed using PCR as described previously [[16], [17], [18]]. PCR products were purified and sequenced by Eurofins MWG Operon (Ebersberg, Germany) for detail genotypic characterization.

### Epidemiological typing by rep-PCR and MLST

The genetic fingerprint of *A. baumannii* isolates was determined by rep-PCR using ERIC1R (5′-ATGTAAGCTCCTGGGGATTCAC-3′) primer. Briefly, amplification was conducted in a total volume of 25 μl containing 2 mM dNTP, 10x PCR buffer, 25 mM MgCl2, 5 U/μl of HotStar taq polymerase (QIAGEN GmbH, Hilden, Germany), primer ERIC1R and 5 μl of the DNA template. Cycling parameters were as follows: 1 min at 94°C, 1 min. at 36°C, and 2 min. at 72 °C for 45 cycles. A final extension at 72°C for 5 min. was performed afterward. The amplified products were visualized on a 1.5 % agarose gel and band patterns were further analyzed visually. In order to explore detailed epidemiological features and sequence types (ST), 15 clinical isolates were selected randomly for MLST analysis according to Pasteur's MLST scheme (http://pubmlst.org/abaumannii/) focusing on 7 standard housekeeping genes (*cpn60, fusA, gltA, pyrG, recA, rplB* and *rpoB)*. Additionally, all environmental (n=10) isolates were also included in the MLST analysis. PCR amplification was carried out in T100™ Thermal Cycler (Bio-rad, USA). PCR products were purified according to the manufacturer's instructions using a QIAquick PCR purification kit (Qiagen, Germany) and then sequenced at Eurofins MWG Operon, Germany. Determination of the sequence type was carried out using the Pasteur MLST Database.

## Results

### Strain distribution and antibiotic resistance profile

In total, 49 clinical isolates and 10 environmental isolates were confirmed as *A. baumannii*. Clinical *A. baumannii* were resistant to ciprofloxacin (100%), imipenem (91%), meropenem (91%), gentamicin (91%), amikacin (87%), and trimethoprim-sulfamethoxazole (61%). The majority (>59%) of the isolates were MDR (resistant to >3 different antibiotic classes). All environmental *A. baumannii* were completely resistant to imipenem, meropenem, gentamicin, amikacin, and ciprofloxacin. All environmental isolates were MDR (resistant to 3–4 different antibiotic classes). The phenotypic diversity of antibiotic resistance among all the clinical and environmental *A. baumannii* is presented in Table 1.

**Table 1:** Phenotypic diversity of antibiotic resistance among clinical and environmental *A. baumannii* isolates

Antibiotic Resistance Phenotypes	No of isolates	Source
IPM-MEM-AK-CN-CIP-SXT	28 (26+2)	Human & Environment
IPM-MEM-AK-CIP-SXT	3	Human
IPM-MEM-AK-CN-CIP	22 (14+8)	Human & Environment
IPM-MEM-CN-CIP	2	Human
CN-CIP-SXT	1	Human
CN-CIP	2	Human
CIP	1	Human

IMP; Imipenem(10μg), MER; Meropenem(10μg), CN; Gentamicin(10μg), AK; Amikacin(30μg), CIP; Ciprofloxacin(5μg) and SXT; Trimethoprim-sulfamethoxazole(25μg).

### Detection of antibiotic resistance and biofilm genes

Genotypic screening of clinical samples revealed the presence of *bla*_*OXA-23*_ in 41 strains and *bla*_*OXA-58*_ in one strain. The genes *bla*_OXA-23_ and *bla*_OXA-58_ are considered as carbapenem resistance markers. The gene *bla*_PER-7_ was present in 6 clinical strains. Other genes detected among the clinical *A. baumannii* isolates were *aac(6′)-Ib-cr* (1/49) and *qnrB1* (7/49) encoding fluoroquinolone resistance, and *armA* (33/49) encoding aminoglycoside resistance. All environmental *A. baumannii* were positive for *bla*_OXA-23_ but no *bla*_OXA-58_ genes were found in the environmental collection. The *bla*_*PER-7*_ was present in 4 environmental strains. Regarding fluoroquinolone resistance markers, only *qnrC1* was found in one environmental strain. The aminoglycoside resistance gene *armA* was present in 8 environmental strains. All tested *A. baumannii* strains were positive for biofilm-related genes; *bap, csuE*, and *pgaB* were found in both clinical and environmental samples.

### Epidemiologic features of A. baumannii

Epidemiological typing by rep-PCR identified 36 different genotypes from the clinical and environmental sets. Isolates differing by one strong band or more were assigned to different genotypes, whereas isolates differing with one weak band from their genotype were assigned to different subtypes. The predominant genotypes were AC and FC. There were some genotypes (AC, BC, DC, FC, HC and PC) that were predominantly circulating only in patients. Some of the *A. baumannii* genotypes were found both in humans and the hospital environment, such as CC, RC, P3, and P6. Notably, environmentally disseminated genotypes RC and P6 were found in the ICU of the hospital. MLST analysis of all clinical and environmental *A. baumannii* isolates (n = 25) revealed 6 different sequence types; ST2 (n = 16), ST10 (n = 2), ST149 (n = 2), ST575 (n = 3), ST1063 (n = 1) and ST1065 (n = 1). ST1063 and ST1065 were novel sequence types assigned by MLST curator. ST1063 and ST1065 have their evolutionary origins from ST149 and ST25 respectively. A detailed epidemiologic and molecular profile is shown in Table 2.

**Table 2:** Genotypic characterization of clinical and environmental *A. baumannii* isolates from Bangladesh

MLST type	rep-PCR profile	Antibiotic resistance genes	Biofilm genes	Source
ST149	CC	*armA+*bla_OXA-23_*+bla*_PER-7_	*bap, csuE, pgaB*	Human
	CC	*armA+*bla_OXA-23_*+bla*_PER-7_	*bap, csuE, pgaB*	Environment (Ventilator)
ST2	P3	*armA*+ bla_OXA-23_	*bap, csuE, pgaB*	Human
	AC	*qnrB1+ armA+* bla_OXA-23_	*bap, csuE, pgaB*	Human
	AC	*qnrB1+ armA+* bla_OXA-23_	*bap, csuE, pgaB*	Human
	BC	*armA+* bla_OXA-58_	*bap, csuE, pgaB*	Human
	BC	*qnrB1+ armA+* bla_OXA-23_	*bap, csuE, pgaB*	Human
	BC	*qnrB1+ armA+* bla_OXA-23_	*bap, csuE, pgaB*	Human
	PC	*armA+* bla_OXA-23_	*bap, csuE, pgaB*	Human
	FC	*qnrB1+ armA+* bla_OXA-23_	*bap, csuE, pgaB*	Human
	WC	*aac(6′)1b-cr+armA+*bla_OXA-23_*+bla*_PER-7_	*bap, csuE, pgaB*	Human
	Z4	*armA+* bla_OXA-23_	*bap, csuE, pgaB*	Environment (Catheter)
	Z4	*armA+* bla_OXA-23_	*bap, csuE, pgaB*	Environment (Ventilator)
	Z3	*armA+* bla_OXA-23_	*bap, csuE, pgaB*	Environment (Bed side table)
	P1	*armA+* bla_OXA-23_	*bap, csuE, pgaB*	Human
	P6	*armA+* bla_OXA-23_	*bap, csuE, pgaB*	Environment (Ventilator)
	RC	*armA+* bla_OXA-23_	*bap, csuE, pgaB*	Environment (Oxygen mask)
	P3	*armA+* bla_OXA-23_	*bap, csuE, pgaB*	Environment (Bed sheet)
ST10	P6	*armA+*bla_OXA-23_*+bla*_PER-7_	*bap, csuE, pgaB*	Human
	QC	*armA+*bla_OXA-23_*+bla*_PER-7_	*bap, csuE, pgaB*	Human
ST575ss	RC	bla_OXA-23_*+bla*_PER-7_	*bap, csuE, pgaB*	Human
	RC	bla_OXA-23_*+bla*_PER-7_	*bap, csuE, pgaB*	Environment (Pillow)
	Z2	*qnrC1+* bla_OXA-23_*+bla*_PER-7_	*bap, csuE, pgaB*	Environment (Sucker)
ST1063	EC	*armA+* bla_OXA-23_*+bla*_PER-7_	*bap, csuE, pgaB*	Human
ST1065	Z1	*armA+* bla_OXA-23_*+bla*_PER-7_	*bap, csuE, pgaB*	Environment (Toilet Sink)

## Discussion

In this study, a majority of the clinical and environmental strains were MDR; displaying resistance to 3–4 different groups of broad spectrum antibiotics. Ciprofloxacin, gentamicin, amikacin and trimethoprim-sulfamethoxazole are extensively used in human medicine [19]. Genotypic analysis revealed the presence of *bla*_*OXA-23*_ and *bla*_*OXA-58*_ genes as carbapenemase-encoding genes. The *bla*_OXA-23_ gene is global disseminated in patients and hospital environments, including Asian countries [3,20]. Thus, *bla*_*OXA-23*_ is widely disseminated in patients and hospital environments of the examined hospitals of Bangladesh. On the other hand, the *bla*_*OXA-58*_ gene has not been able to spread as widely in Bangladesh as it has in other Asian countries [20]. The *bla*_PER-7_ gene is plasmid-associated and has been reported in clinical isolates in the United Arab Emirates [2]. The *bla*_PER-7_ -carrying *A. baumannii* isolates are reported in Bangladesh for the first time and found in both clinical and environmental isolates, indicating that the gene could be widespread in Bangladesh. All clinical and environmental isolates were phenotypically resistant to ciprofloxacin and few of their corresponding genotypes were found through molecular approach; *qnrB1, aac(6′)-Ib-cr* and *qnrC1*. Since all *A. baumanni* isolates were phenotypically resistant to fluoroquinolones, and genotyping screening revealed few plasmid-mediated fluoroquinolone resistance determinants, it is likely that the remaining isolates had chromosomal resistance mechanisms [21]. In this study, *armA* was the only aminoglycoside resistance gene found in the majority of the aminoglycoside resistant isolates. Hospital dissemination of *armA* in relation to high levels of aminoglycoside resistance in *A. baumanni* was reported from Asia [22]. MDR *A. baumannii* isolates are widespread in both the clinical and environmental settings of Bangladeshi hospitals, therefore, leaving limited options for the treatment of *A. baumannii* infections in Bangladesh.

Studies suggested that antibiotic resistant bacteria are highly associated with biofilm formation capacities, and biofilm related genes of *A. baumannii* including *bap, csuE*, and *pgaB* were responsible for biofilm development [6]. In this study, both clinical and environmental isolates with known sequence types were carrying all biofilm-associated genes investigated: *bap, csuE*, and *pgaB*. This is the first study reporting the presence of biofilm associated genes in the *A. baumannii* isolates from patients and hospital environments in Bangladesh.

The epidemiological typing identified some dominant clinical genotypes found in the ICU of the hospital. There were some other minor genotypes found both in ICU and non-ICU hospital environments including medical devices, which indicates cross contamination of the ICU from environmental sources or vice versa. The dissemination and dominance of *A. baumannii* clonal type ST 2 was reported in several hospitals globally [1]. MLST indicated that *A. baumannii* ST2 (international clone 2) was disseminated in hospital settings of Bangladesh. A majority of the Bangladeshi *A. baumannii* ST2 were associated with *bla*_*OXA-23*_ and *armA* genes, and this strain type has a global spread that includes other Asian countries like Vietnam [23]. In the present study, ST10 and ST575 were found to be common after ST2 in this Bangladeshi hospital. In this study, *A. baumannii* ST575 was the second most dominant sequence type, found in both clinical and environmental settings of Bangladesh, which was reported previously as an emerging strain in Vietnam [23]. The sharing of common ST clusters between clinical and environmental strains indicated cross contamination and suggests problems with infection control practices. The presence of these *A. baummanii* STs in surfaces of the hospital could potentially be a source of hospital-acquired infections.

Hygiene practices are very poorly managed or not managed at all in a majority of Bangladeshi hospitals due to limited economic and technical resources (personal communication). A study from Bangladesh reported an abundance of ESBL-producing bacteria in patients and hospitals environments resulting from mismanagement of hospital wastes and poor hygiene practices [7]. Improper hospital hygiene, overcrowding, misuse of antibiotics, and poor infection control strategies seem to be contributing factors to this situation in Bangladeshi hospitals. Thus, the spread of carbapenem resistant clones is an indication of the concerning situation in major Bangladeshi hospitals.

## Conclusion

Carbapenemase-producing MDR *A. baumannii* capable to develop biofilm were found in patients and in environmental cultures from Bangladeshi hospitals. This study explored the dissemination of carbapenem-resistant *A. baumannii* clones in hospital settings in Bangladesh and indicate the possible clonal spread. This warrants futher detailed investigation by high-resolution typing method like whole genome sequencing. There is an urgent need to establish nationwide antimicrobial resistance surveillance and infection control strategies in Bangladeshi hospitals.

## Credit author statement

**Refath Farzana**; Methodology, Investigation, Formal analysis, Writing- Original draft. **Göte Swedberg**; Formal analysis, Writing - Review & Editing. **Christian G. Giske**; Formal analysis, Writing - Review & Editing. **Badrul Hasan**; Conceptualization, Funding acquisition, Supervision, Investigation, Methodology, Formal analysis, Writing - Review & Editing.

## Conflict of interest

No competing financial interests exist.

## Funding

This work was financially supported by Emil & Ragna Börjeson Foundation (10.13039/501100007051Uppsala University), ISID-ESCMID fellowship scheme (10.13039/100003024International Society for Infectious Diseases), 10.13039/100010823Tore Nilson's Fund for Medical Research (2014-00083) and 10.13039/100007435Åke Wibergs Foundation (M14-0291).
